# Is Matang Mangrove Forest in Malaysia Sustainably Rejuvenating after More than a Century of Conservation and Harvesting Management?

**DOI:** 10.1371/journal.pone.0105069

**Published:** 2014-08-21

**Authors:** Arnaud Goessens, Behara Satyanarayana, Tom Van der Stocken, Melissa Quispe Zuniga, Husain Mohd-Lokman, Ibrahim Sulong, Farid Dahdouh-Guebas

**Affiliations:** 1 Laboratory of Systems Ecology and Resource Management, Université Libre de Bruxelles - ULB, CPI 264/1, Brussels, Belgium; 2 Mangrove Research Unit (MARU), Institute of Oceanography and Environment (INOS), Universiti Malaysia Terengganu - UMT, Kuala Terengganu, Malaysia; 3 Laboratory of Plant Biology and Nature Management, Vrije Universiteit Brussel - VUB, Brussels, Belgium; Centro de Investigación y de Estudios Avanzados, Mexico

## Abstract

Matang Mangrove Forest Reserve (MMFR) in Peninsular Malaysia is under systematic management since 1902 and still considered as the best managed mangrove forest in the world. The present study on silvimetrics assessed the ongoing MMFR forest management, which includes a first thinning after 15 years, a second thinning after 20 years and clear-felling of 30-year old forest blocks, for its efficiency and productivity in comparison to natural mangroves. The estimated tree structural parameters (e.g. density, frequency) from three different-aged mangrove blocks of fifteen (MF15), twenty (MF20), and thirty (MF30) years old indicated that *Bruguiera* and *Excoecaria* spp. did not constitute a significant proportion of the vegetation (<5%), and hence the results focused majorly on *Rhizophora apiculata*. The density of *R. apiculata* at MF15, MF20 and MF30 was 4,331, 2,753 and 1,767 stems ha^−1^, respectively. In relation to ongoing practices of the artificial thinnings at MMFR, the present study suggests that the first thinning could be made earlier to limit the loss of exploitable wood due to natural thinning. In fact, the initial density at MF15 was expected to drop down from 6,726 to 1,858 trees ha^−1^ before the first thinning. Therefore the trees likely to qualify for natural thinning, though having a smaller stem diameter, should be exploited for domestic/commercial purposes at an earlier stage. The clear-felling block (MF30) with a maximum stem diameter of 30 cm was estimated to yield 372 t ha^−1^ of the above-ground biomass and suggests that the mangrove management based on a 30-year rotation is appropriate for the MMFR. Since Matang is the only iconic site that practicing sustainable wood production, it could be an exemplary to other mangrove locations for their improved management.

## Introduction

Mangrove forests are considered as one of the most productive ecosystems in the world and have a well-established ecological, economic and cultural importance [1], [2], [3], [4], [5], [6], [7], [8], [9], [10]. However, the constant pressure exerted by anthropogenic (more than natural) events is responsible for its decline at a faster rate than that of tropical rainforests [11], [12], [13], [14], [15]. Along with mangrove cover depletion, the loss of its biodiversity and economic value are the other perturbing issues [8], [16]. In particular, mangroves should be treated carefully without underestimating their role for local livelihoods, and see their long-term benefits reach future generations via appropriate conservation and management practices [17], [18], [19].

**Figure 1:**
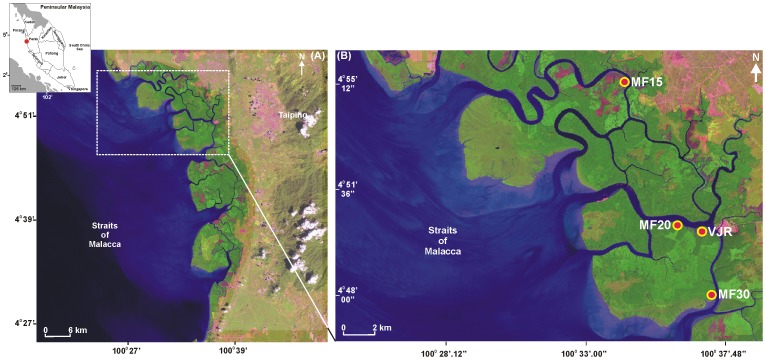
Matang Mangrove Forest Reserve in the state of Perak on the West coast of Peninsular Malaysia (A) (dotted square represents the study zone); (B) Location (yellow circle with red dots) of the Virgin Jungle Reserve (VJR) and the Managed Mangrove Forest (MF with 15, 20 and 30 year old vegetation) blocks considered for silvimetric measurements in the present study (image source: Landsat 7 dated 27 Dec 1999 from the NASA's Earth Observatory).

**Figure 2:**
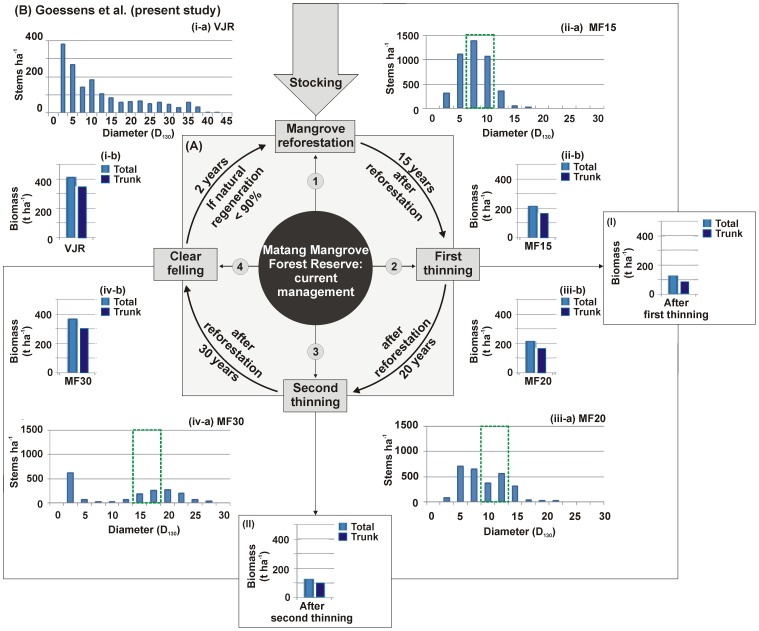
Schematic representation showing the Matang mangrove management system (A); (B) the present observations on *Rhizophora apiculata* – (a) stem size (D_130_) and, (b) biomass distribution at different forest blocks: (i) VJR: Virgin Jungle Reserve, (ii) MF15: Managed Forest at 15 years old, (iii) MF20: Managed Forest at 20 years old and, MF30: Managed Forest at 30 years old; B (I–II): the computed biomass of *R. apiculata* after the first and second thinnings. The green coloured rectangle (dashed box) in B (ii-a to iv-a) shows the expected/required stem size by the State Forestry Department of Perak.

The Matang Mangrove Forest Reserve (hereafter referred to as ‘MMFR’), located on the northwest coast of Peninsular Malaysia (Fig. 1), is under concerted scientific management since the beginning of the 20^th^ century and still considered as the best managed mangrove forest in the world [2], [20]. The management here is based on a 30-year rotation cycle with two artificial thinnings in 15 and 20-year old blocks [21] (Fig. 2A). These artificial thinnings are based on a spacing technique called the ‘stick method’: whereas the first thinning is carried out for any tree within a 1.2 m radius of the selected central tree (measured by a stick this long), the second thinning will be done for any tree within a 1.8 m radius. The aim of these thinnings is not only to obtain poles [22], but also to promote better growth of the remaining trees [23]. After the clear felling in a 30-year old block (for charcoal production), the area is replanted with the seedlings of *Rhizophora apiculata* Blume at 1.2 m and *R. mucronata* Lamk. at 1.8 m intervals [21]. However, the local management authorities at Matang are still focused on an improved productivity because of declining yields in recent years [24]. While management decisions are often based on poor or incomplete information [25], the State Forestry Department of Perak is devoted to accomplish scientific studies, and update, if necessary, the existing production and conservation policy at Matang.

Although few studies have attempted to validate the 30-year forest rotation plan for Matang, the availability of (field-based) tree structural measurements was a constraint. For example, the first and only published study on silviculture vis-à-vis management system was Gong and Ong in 1995 [26]. They have studied the demography of the forest by analyzing size distribution and biomass in different aged blocks. However, in view of their data collected in November 1980 (from just four adjacent 10 m×10 m plots), they have expressed the low reliability of their results and recommended further studies with a more intensive regime. Apart from the literature on plant biomass and nutrient fluxes [27], [28], [29]; sediment and recycling organic matter [30], [31], [32]; canopy gaps and regeneration [23], etc., and despite its exemplary potential, there was no (published) scientific field-based study on the tree structural assessment in relation to conservation and management guidelines at the MMFR before the present study. Even the recent innovative paper by Fontalvo-Herazo et al. [33] on silviculture management using individual-based model relied upon the 20 year-old literature data from Gong and Ong [26]. Hence, there is a need for understanding whether or not the harvesting regime leads to a sustainable rejuvenation i.e., an efficient and value-added long-term use of the forest resources while having a minimum environmental impact.

The present paper aimed at sustainable rejuvenation validation in Matang, both from an ecological and a silvicultural point of view. In addition, the assessment of silvimetric parameters (juvenile, young and adult tree density, vegetation biomass) from different aged forest blocks offers field-based recommendations to the local authorities for a better mangrove management. Considering that mangroves occur in 123 countries on all continents with tropical climates, and that no other mangrove forest on the planet has been managed for over a century (not even for half that period) our study also serves as a reference for long-term mangrove forest management. It also opens doors to investigate mangrove charcoal trade and its ecological and socio-economic implications.

## Materials and Methods

### Study area

The MMFR covers 40,288 ha in the state of Perak (4°45′N, 100°35′E) (Fig. 1) and is a typical riverine mangrove forest in Malaysia [21]. The reserve is shaped like a crescent moon along the 51.5 km coastline from Kuala Gula in the north to Bagan Panchor in the south. The major portion of the reserve lies on seven deltaic islands separated by a network of creeks and canals [34]. We recall that this MMFR is under sustainable management by the State Forestry Department of Perak for timber production since 1902 [21], [23]. As required by the Malaysian National Forestry Policy, the Matang management also has other objectives such as shoreline protection from coastal erosion, to assure functionality of the forest as breeding/nursery ground and wildlife habitat, and as support for forest conservation, research, education and ecotourism [21], [34].

The latest version of the Matang management plan covers the period 2010–2019, and is also based on a 30-year rotation cycle [35]. It should be noticed that the MMFR has four management zones namely, ‘protective’, ‘productive’, ‘restrictive productive’ and ‘unproductive’ forests of which the productive zone got 110 compartments/blocks with different aged *Rhizophora* stands to implement both the thinning and the clear-felling operations on a regular basis. This working plan also indicated several research needs such as monitoring of the forest transitions (i.e. from *Avicennia* to *Rhizophora*, *Rhizophora* to dryland and reversion from dryland to *Rhizophora*), the scattered circular zones of dead trees (due to lightning strikes), the trees collapsing onto navigating boats (buffer zones), and the pharmacological use of mangrove species. Along with *R. apiculata* and *R. mucronata*, the other mangrove species found in the area were *Avicennia officinalis* L., *Bruguiera gymnorrhiza* (L.) Lamk., *B. parviflora* Wight and Arnold ex Griffith, *B. cylindrica* (L.) Blume, *Ceriops tagal* (Perr.) C.B. Rob., *Excoecaria agallocha* L., and *Sonneratia alba* J. Smith.

MMFR is influenced by an equatorial climate (warm and humid) with a mean annual temperature of 23.7–33.4°C and humidity of 76.5–83.5%. The rainfall, varying between 2000 and 3000 mm, occurs throughout the year [24]. The tides are semi-diurnal with an amplitude in the range of 1.60–2.98 m [36].

### Fieldwork and data analysis

The present study was carried out from February to April, 2011. To obtain the tree structural parameters (e.g. girth, height), both the Virgin Jungle Reserve (VJR: 4°50′3″N, 100°37′0″E), and the Managed Forest (MF) were visited with a prior permission from the State Forestry Department of Perak. While the former has not been exploited for at least 80 years, and therefore considered ‘natural’, the latter comprises the productive mangrove forest blocks with three different ages of fifteen (MF15: 4°55′16″N, 100°34′9″E), twenty (MF20: 4°50′22″N, 100°36′12″E), and thirty (MF30: 4°48′10″N, 100°37′25″E) years old (Fig. 1B). Tree girth (G_130_) was measured at 130 cm height above the ground and along the stem using a measuring tape, while the tree height was estimated with the help of a MDL LaserAce 300 (accuracy: 10 cm). For tree measurements, the suggestions offered by Dahdouh-Guebas and Koedam [37] were followed. It is worth mentioning that there are also multiple-stemmed trees (MST) in the forest. Therefore we estimated both the number of trees ha^−1^ considering the MST as a unique tree and, the number of stems ha^−1^ considering each stem of the MST as a separate tree. Although single and MST are commonly found in the mangroves [37], there was not much debate on this issue and the related research, such as the valuable work of Gong and Ong [26], did not refer to it.

At each forest block, two parallel transects (50 m apart) were laid from the waterfront to the inland mangrove and we counted as well as measured the trees within 10 m×10 m plots at 50 m intervals along each transect. Altogether 67 plots (VJR: 21, MF15: 16, MF20: 15 & MF30: 15) were sampled. In addition to adult trees (≥1.3 m height with a stem diameter D_130_≥2.5 cm or G_130_≥8 cm) measurements, the data (nos.ha^−1^) on mangrove juveniles ( = fallen propagules and saplings with three leaf pairs or less), and young trees (<1.3 m height with a D_130_<2.5 cm or G_130_<8 cm) (divisions comparable to Kairo et al. [38]), were also collected to examine the natural recruitment in the blocks. The taxonomic identification of mangroves was based on using Tomlinson [39]. A handheld global positioning system (Garmin, GPS III) was used for navigation.

Tree density (no. ha^−1^), basal area (m^2^ ha^−1^), relative density (%), relative dominance (%), relative frequency (%), and species' individual rankings were estimated by following the standard protocols [37], [40], [41], [42], [43]. In view of the prevailing MST in each forest block, the actual density of trees was calculated by the following equation:
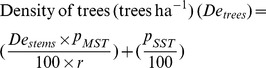
(1)where *De_stems_* is the density of stems (see Eq. 2); *p_MST_* – the percentage of multiple-stemmed trees; *r* – mean number of stems per multiple-stemmed tree; *p_SST_* – the percentage of single-stemmed trees.

At the same time, the possible loss of stems (*Q*) before the first thinning at MF15 was also estimated using the equation below:
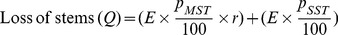
(2)where *E* is the expected loss of tree density (see eq. 1 for other terms).

And finally, the trunk and above-ground biomass of *Rhizophora* trees were calculated through the allometric relationships published by Ong et al. [44] for Matang:
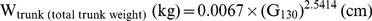
(3)

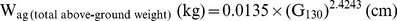
(4)


Since the managed forest blocks MF15 and MF20 are scheduled for their first and second thinnings in the year 2011 (not yet started at the time of this study), we also tried to compute the density as well as biomass of *R. apiculata* that is likely going to be present in the two blocks after thinning. For this purpose, we followed Ong [22] who has estimated that 35 to 50% of the trees will be removed after each thinning process. To simplify, it has been averaged to 40% reduction at each block which also coincides with the self-thinning percentage (40%) of Gong and Ong [26]. These projected values are supposed to complement the field data collected by the forest authorities after thinnings, and provide an indication on the forest structure intervened with the ongoing silvicultural practices. However, the authors found that there is no such practice of taking tree structural measurements officially and hence the present results were compared only with the available literature on Matang management [26], [33].

### Statistical analysis

Tree structural variables were estimated using the standard as well as above mentioned equations in Microsoft Excel. In addition, the non-parametric one-way analysis of variance (One-way ANOVA) was used to find differences between the stem size (D_130_) distribution and the tree density counts at ‘virgin’ and ‘managed’ forest blocks (using GraphPad Prism *v*.5 software). The distance-based redundancy analysis (dbRDA) - a method of constrained ordination which can display the relationships among samples points from a fitted model [45], [46], [47], was used to know the percentage (%) variation between the virgin and the managed mangrove blocks in relation to their juvenile, young and adult vegetation (using PRIMER *v*.6.1.12 software). In this context, the analyses were conducted on a Bray-Curtis similarity matrix calculated from the vegetation counts (root-transformed data for approximating poisson to normal distribution).

## Results

### Stand structure

**Figure 3:**
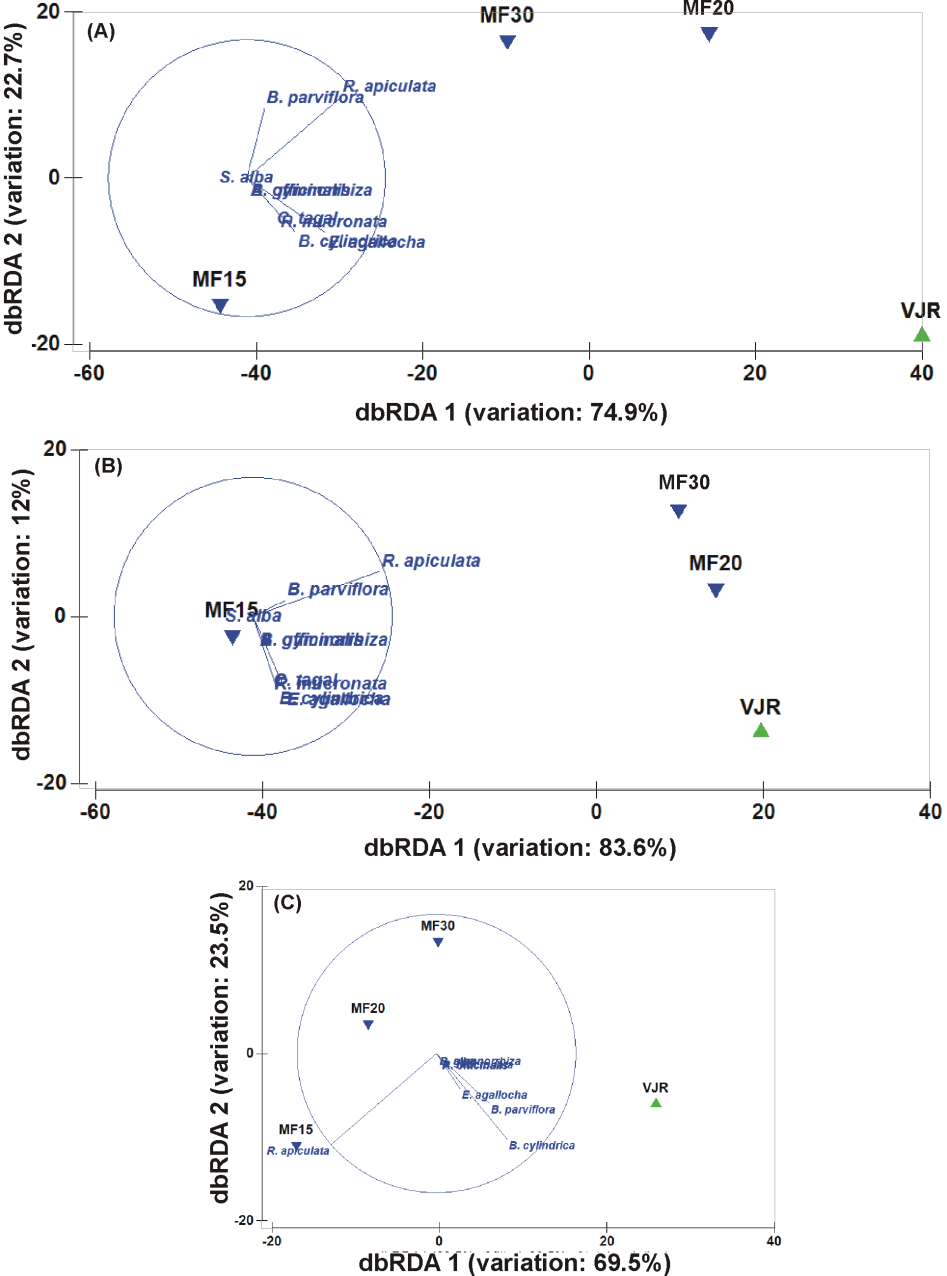
The distance-based redundancy analysis (dbRDA) showing variations between virgin and managed mangrove forest blocks in relation to their – (A) juvenile, (B) young and, (C) adult vegetation at the Matang Mangrove Forest Reserve. While density of the juvenile and the young vegetation was estimated for nos. ha^−1^, the adult tree density was estimated for no. stems ha^−1^ (VJR: Virgin Jungle Reserve; MF15, MF20 and MF30: Managed Forest blocks at 15, 20 and 30 years old) (circles in all panels represent correlation circles, and the orientation of mangrove species' lines approximate their correlation to the ordination axes).

In all forest blocks, *R. apiculata* was dominant whereas *B. parviflora*, *B. cylindrica*, *R. mucronata* and *E. agallocha* did not constitute more than 5% of the total density (Table 1). Hence the results of this study were focused majorly on *R. apiculata*. In fact, the variability between plots was also high and in most cases the species like *R. mucronata*, *E. agallocha* and *B. cylindrica* occurred only in single plots (Table 1). The computed values show that the density of *R. apiculata* would decrease from 4,331 to 2,599 stems ha^−1^ at MF15 after the first thinning and, from 2,753 to 1,652 stems ha^−1^ at MF20 after the second thinning (Table 1). The (total) basal area was ranged between 27.11 and 37.62 m^2^ ha^−1^ in the managed forest blocks of MF20 and MF30, respectively. In the case of VJR, the stem density was 2,352 ha^−1^ (Table 1), with a basal area of 42.17 m^2^ ha^−1^. Followed by *R. apiculata*, *B. cylindrica* and *B. parviflora* were the most frequently seen species in this block (Table 1). The proportion of MST was higher at MF15 (78%) followed by MF20 (23%) (Table 1). The distance-based redundancy analysis (dbRDA) (Fig. 3) indicated that the variance between VJR and MF blocks (i.e. MF15, MF20 and MF30) was higher for young trees (variation along axis-1: 83.6%), than to juvenile (variation: 74.9%) and/or adult (variation: 69.5%) vegetation. It also shows a decreasing trend of adult *R. apiculata* density from MF15>MF20>MF30 to VJR (Fig. 3C).

**Table 1:** Adult tree density (stems ha^−1^) and frequency (%) at Matang Mangrove Forest Reserve.

Species	VJF	MF15	Thinning-I	MF20	Thinning-II	MF30
*Rhizophora apiculata*	1,633	4,331	2,599	2,753	1,652	1,767
	(100%)	(100%)		(100%)		(100%)
*R. mucronata*	10*	44*	26	-	-	-
	(5%)	(12%)				
*Bruguiera parviflora*	271	13*	-	20	-	87
	(33%)	(6%)		(13%)		(47%)
*B. cylindrica*	291	6*	-	-	-	-
	(38%)	(6%)				
*B. gymnorrhiza*	5*	-	-	-	-	-
	(5%)					
*Excoecaria agallocha*	119	-	-	33*	-	-
	(14%)			(7%)		
*Avicennia officinalis*	19	-	-	-	-	-
	(10%)					
*Sonneratia alba*	5*	-	-	-	-	-
	(5%)					
Total:	2,352	4,394	-	2,807	-	1,853
MST proportion (%)	5.25	77.82		22.98		5.21
No. stems per MST	2.25	2.71		2.26		2.30

The values under thinnings I and II are the computed stem density which is likely to be present after the thinning events at MF15 and MF20. VJR is Virgin Jungle Reserve; MF15, MF20 and MF30 are the Managed Forest blocks at 15, 20 and 30 years old. MST is multiple-stemmed tree. Except *Rhizophora*, all other species encountered during inspection visits and/or thinning operations at the managed forest blocks will be clear-felled.

*found only at single plot.

### Size distribution

At VJR, although the majority of the *Rhizophora* trees come under adult vegetation category, the density of stems holding 2.5–10 cm diameter (D_130_) was high (971 stems ha^−1^) (max. diameter, 42.5 cm) (Fig. 2B-i-a). In the case of managed mangrove forests, the MF15 had more stems with 5–12.5 cm diameter (3,587 stems ha^−1^) (max. diameter, 20 cm) (Fig. 2B-ii-a), while MF20 with 5–15 cm (1,733 stems ha^−1^) (max. diameter, 25 cm) (Fig. 2B-iii-a), and MF30 with 15–22.5 cm (906 stems ha^−1^) (max. diameter, 30 cm) (Fig. 2B-iv-a). At the above given ranges of stem size (D_130_) distribution, the tree densities in the VJR and the MF blocks were significantly different (One-way ANOVA, *F* = 8.7, *R^2^* = 0.67, *P* = 0.002).

### Natural recruitment

Data on young and juvenile vegetation at the managed forest blocks also revealed the higher abundance of *R. apiculata* (Table 2). Among the managed blocks, MF20 supported maximum number of young (4,227 ha^−1^) and juvenile (2,867 ha^−1^) vegetation.

**Table 2:** Density (no. ha^−1^) and frequency (%) of the juvenile and young vegetation at Matang Mangrove Forest Reserve.

	VJR				MF15				MF20				MF30			
	Juvenile		Young		Juvenile		Young		Juvenile		Young		Juvenile		Young	
Species	Den	Freq	Den	Freq	Den	Freq	Den	Freq	Den	Freq	Den	Freq	Den	Freq	Den	Freq
*Rhizophora apiculata*	4,238	91	2,795	86	94	56	138	31	2,620	100	2,973	100	1,040	93	3,453	100
*R. mucronata*	190	14	91	14	-	-	-	-	-	-	-	-	-	-	-	-
*Bruguiera parviflora*	1,286	24	367	48	19	12	125	50	200	13	967	13	60	13	347	20
*B. cylindrica*	571	19	143	29	-	-	-	-	-	-	-	-	-	-	-	-
*B. gymnorrhiza*	-	-	5	5	-	-	-	-	-	-	-	-	-	-	-	-
*Excoecaria agallocha*	-	-	248	10	-	-	13	6	47	7	287	7	-	-	-	-
*Avicennia officinalis*	-	-	5	5	-	-	-	-	-	-	-	-	-	-	-	-
*Sonneratia alba*	-	-	-	-	-	-	-	-	-	-	-	-	-	-	-	-
*Ceriops tagal*	857	5	81	5	-	-	-	-	-	-	-	-	-	-	-	-
Total:	7,142		3,735		113		276		2,867		4,227		1,100		3,800	

VJR, MF15, MF20 and MF30 are the Virgin Jungle Reserve and the Managed Forest blocks at 15, 20 and 30 years old. Den is density, Freq is frequency.

### Vegetation biomass

The trunk and above-ground (total) biomass at MF15 and MF20 were almost equal (216–217 t ha^−1^), whereas MF30 had 372 t ha^−1^ with a difference of 43 t ha^−1^ in relation to VJR (i.e. 415 t ha^−1^) (Figs. 2B-i-b to 2B-iv-b). In the case of MF15, the biomass after the first thinning is expected to come down from 216 to 130 t ha^−1^ and, at MF20 after the second thinning it could reduce from 217 to 130 t ha^−1^ (Figs. 2B-I and 2B-II).

## Discussion

### Initial stocking

Since the density of *R. apiculata* at MF15 (4,331 ha^−1^) (Table 1) was a stem-based count, it does represent the number of stems exploitable as poles, but it does not reflect the actual number of trees planted/present in this block. Therefore, according to the fact that 78% of the trees at MF15 are MST (with a mean no. of 3 stems per tree) (Table 1), the actual density of *R. apiculata* trees is expected to be *ca.* 1,858 trees ha^−1^.

If the mangrove management objective is only to have a dense forest cover (and an ecologically functional forest), all naturally recruited (mangrove) seedlings should be considered for effective stocking [38]. However, if forests are meant for commercial exploitation (also with selective species) plantations such as in MMFR seem to be the rule. Under the actual management system at Matang, artificial planting is carried out within two years after clear felling (at MF30), provided the density of the natural recruitment in the clear-cut areas is less than 90% [26], [35]. However, in most cases, afforestation was said to be necessary (pers. comm. with the authorities of the State Forestry Department of Perak) due to the limited number of propagules stranding in the clear-felled locations after hydrochorous dispersal. The recommended as well as the implemented space for artificial (*R. apiculata*) regeneration was 1.2 m×1.2 m, which allows a planting density of 6,726 seedlings ha^−1^ [48]. This information enables us to compare the actual planting density with the trees available after 15 years of management (i.e. 1,858), to conclude that there was a loss of juvenile, young and/or adult trees of as much as 4,868 individuals (72%). In natural environments, the juvenile/young/adult tree ratios reflect particular stages in distribution and survival [16]. While juveniles represent a possibility for a species to reach a particular location through simple fall and/or hydrochory [49], [50], [51], young trees represent survival from propagule predation [52], [53], [54], and adult trees represent survival from unfavorable environmental conditions [53], [55]. Since MF15 is an afforested block, the loss of vegetation could be due to the above mentioned factors as well as natural thinning. Meanwhile, the natural recruitment, playing an important role for the rise of seedlings at each forest block (Table 2), should not be ignored. Therefore, in agreement with Gong and Ong [26], to counter propagule loss, artificial regeneration should be conducted if the naturally recruited density is less than 50%, instead of 90%. In addition, we propose the space for artificial regeneration (1.2 m×1.2 m) to be increased not only to reduce the loss of propagules due to competition, but also to allow the trees to better grow and develop. However, in view of the species planted as well as naturally recruited (with different growth patterns) over the period of time, further studies and/or field-based experiments are necessary to determine the most appropriate space for this artificial regeneration. By looking at this outcome, the other mangrove locations elsewhere may also follow the suggestion of increased space for their plantation efforts.

### Stand density

With the ongoing management at the MMFR, except for *Rhizophora*, all other species encountered during inspection visits and/or thinning operations at the managed forest blocks are clear-felled [35]. Therefore the productive forest areas are mostly composed of *Rhizophora* trees [23]. At the time of first thinning in MF15, mangrove stems are expected to reach to a diameter of 7.5–10 cm [21]. However, taking into account the loss of vegetation (4,868 nos.) and the present status of MST (78%), there could be a loss of 11,352 stems ha^−1^ in the 15-year old forest block. Therefore, implementation of the first thinning before a stand age of 15 years may help reducing loss of exploitable biomass due to natural thinning or unfavourable conditions (as explained in the previous section). In addition, the smaller-sized diameter poles resulting from early thinning could be used for domestic or commercial purposes, including firewood or white charcoal production [35]. Earlier, also Gong and Ong [26] suggested more frequent thinning events at shorter intervals. It was further supported by Fontalvo-Herazo et al. [33] who used the pattern oriented modeling (POM) in KiWi to reproduce forest (i.e. *R. apiculata*) density and size class distribution, and thereby the selection of thinning strategies within the 30-year harvesting cycle. Among the four tested types of thinning activities, Fontalvo-Herazo et al. (op. cit.) found that a management plan with three artificial thinnings at the age of 7, 13 and 20 years was most productive. However, the legitimacy of these predictions is known only when they are tested in the field. The raised densities of *R. apiculata* after the first and second thinnings in the present study (Table 1) are attributable to young vegetation present in both forest blocks (i.e. MF15 and MF20) (Table 2), but beyond the area cleared by the stick method.

**Figure 4:**
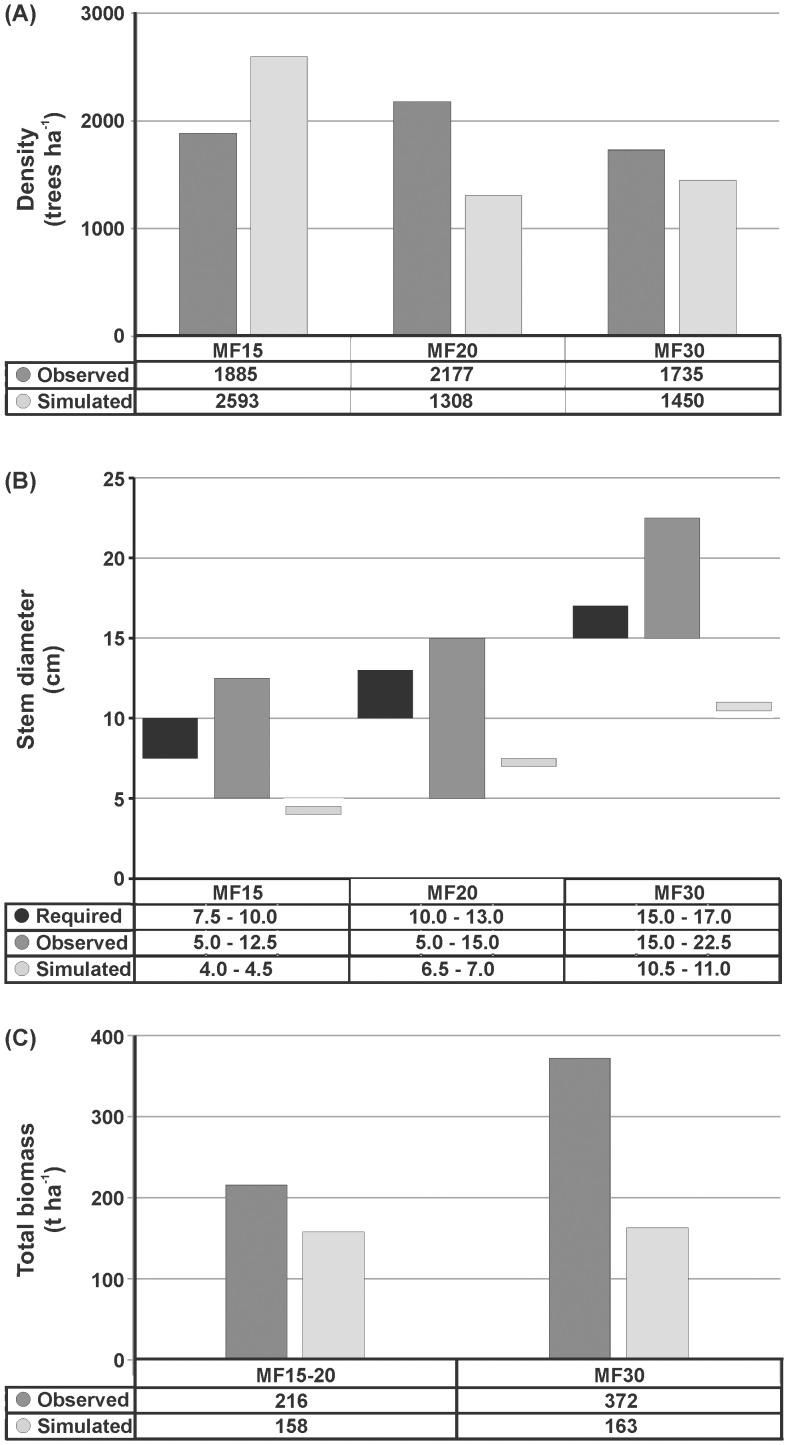
A comparative account on – (A) tree density, (B) Stem diameter (D_130_) and, (C) harvestable (total) biomass range, as observed by the field- and the simulation-based investigations at Matang Mangrove Forest Reserve (MF15, MF20 and MF30: Managed Forest blocks at 15, 20 and 30 years old). While ‘observed’ stands for the present (field-based) study, ‘simulated’ indicates the results obtained from Fontalvo-Herazo et al. (2011). Similarly, ‘required’ is the stem size as expected by the State Forestry Department of Perak.

In addition, the tree density values in the present study were found to be different from the simulation-based predictions by Fontalvo-Herazo et al. [33] (Fig. 4A): the density was more at MF15 (2,593 instead of 1,885 trees ha^−1^ predicted), and less at both MF20 (1,308 instead of 2,177 trees ha^−1^ predicted), and MF30 (around 1,450 instead of 1,735 trees ha^−1^ predicted) forest blocks. Also, the computed densities of *R. apiculata*, after the first and second thinnings, were dissimilar. These discrepancies are nothing but due to two different approaches (field-based and literature-based), limited data available for a precise parameterization in mangrove model, different stocking density and reduction percentages, etc.

### Size distribution

Despite the fact that age and growth relationship can be well established for afforested forests than for natural, the literature on mangrove stem size distribution in relation to age is still limited [38], [56], [57], [58]. Coinciding with the (Matang) Forest Department's required stem size (D_130_) of 7.5–10 cm before first thinning, the majority of the *Rhizophora* stems at MF15 have had 5–10 cm diameter (Fig. 2B-ii-a). A similar trend was also observed in Kenya where 12-year old *R. mucronata* stands, though younger by 3 years, showed a stem size range between 2.5 and 12.4 cm [58]. In the case of the second thinning at MF20, where the actual requisite of stem size was 10–13 cm [21], the stems had grown to a diameter of up to 22.5 cm (Fig. 2B-iii-a). In addition, the prevailing high density of stems with 5–10 cm diameter at MF20 was possibly due to non-removal of the young trees at the time of first thinning. Finally, in the 30-year old forest block (MF30), the diameter reached by the stems (i.e. 15–25 cm) was higher than the required size (15–17 cm) (Fig. 2B-iv-a). One possible reason for this situation is that this forest block may be older than expected. According to local forest authorities, the agenda for thinning/clear-felling cannot be respected sometimes due to administrative difficulties (e.g. work tender finalization, officers' availability, etc.) and hence they conduct the thinning or felling activities earlier or later than scheduled. Another reason could be that the first and second thinnings in this forest block had been very intensive to allow the remaining trees to grow better. The role of natural recruitment, with numerous stems holding 2.5 cm diameter (must have originated from MF20, Table 2), was also evident in this block (Fig. 2B-iv-a). The decreased stem densities are balanced by an increasing diameter (D_130_) between MF15 and MF30 (Figs. 2B-i-a to 2B-iv-a), which is reassuring in the light of a steady-state mangrove resource management [59], but expected to be less functional and less resilient ecologically.

On the other hand, the stem diameter (D_130_) observed by both simulated- and field-based investigations was different (Fig. 4B), possibly due to the lack of a required stem size parameterization in the mangrove model [33] as well as the reasons mentioned above under the ‘Stand density’.

### Natural recruitment

The successfully reforested mangrove sites would be able to improve local hydrodynamics and soil physicochemical conditions that in turn can enhance the natural colonization of both plants and animals [1], [55], [56], [57], [60], [61]. In fact, the witnessed secondary succession of non-planted mangrove species into the managed forest blocks at Matang (Table 2) suggests an effective reforestation such as in Gazi Bay in Kenya [56], [57], [58]. However, the lack of persistence of these recruits into the older vegetation layers (due to the thinning practices) makes it evidently less exemplary as a ‘natural’ forest. Based on density as well as frequency of juvenile and young vegetation at the managed forest blocks (Table 2), MF30 (clear-felling block) is still supporting 4,493 *R. apiculata* individuals (juvenile+young), though it was less than the recommended initial (stocking) density of 6,726 no. ha^−1^ [48]. Despite the fact that natural recruitment should be also considered as a key factor in mangrove restoration [57], [61], it is perhaps difficult to apply of the ongoing MMFR management. Hence, most of the propagules, seedlings and young trees naturally recruited or regenerated at MF30 are lost during the clearing operation [26]. However, post-clearing habitat recovery via natural recruitment (propagule stranding) can still be a pre-planting option.

Next to *R. apiculata*, *B. gymnorrhiza* was abundant and had a chance to grow as co-dominant species in the vicinity (Table 2). However, its young tree density which decreased from 45 to 9% between 15 and 30-year old blocks was due to its removal during the first and second thinnings. On the other hand, *E. agallocha* never represented more than 6.5% of the total density and thus did not constitute an effective competitor for *R. apiculata*, which is not surprising as their optimal elevations within the intertidal zone are different [16], [54]. The lower density of juvenile (113 nos. ha^−1^) and young (276 stems ha^−1^) vegetation at MF15 has made this block distinct from other blocks (Fig. 3A–B). Overall, the juvenile/young vegetation data analysis based on *R. apiculata* as well as other mangrove species (i.e. *A. officinalis*, *B. cylindrica*, *B. gymnorrhiza*, *B. parviflora*, *C. tagal*, *E. agallocha*, *R. mucronata* and *S. alba*) was found to be important for testing how ecologically close the VJR and the MF blocks in terms of their species composition/abundances. The present observations are however in contrast to Fontalvo-Herazo et al. [33] whose model did not allow natural recruitment or self-thinning to happen in both MF15 and MF20 stands.

### Biomass increment

Being the richest carbon pool in the world [62], mangrove forests are important for climate change mitigation via Reduced Emissions from Deforestation and Degradation (REDD+) [63]. However, in view of the long-lasting man-mangrove linkage on one hand and the increased population/over exploitation of the resources on the other, the sustainable management of these forests should be a priority [2], [8], [11], [17].

Gong and Ong [28] compared the biomass of a virgin mangrove stand with the second generation yields of 1967–69 and 1970–77 in the MMFR. While the difference of 163 t ha^−1^ between virgin and managed forest blocks was justified because of the 50–70 years older trees at VJR, they also reported a decline of 22 t ha^−1^ in between the two i.e., 1967–69 and 1970–77 managed blocks. In order to increase the biomass, Gong and Ong (op. cit.) have suggested implementing an earlier silvicultural thinning (for the removal of non *Rhizophora* trees) at the age of 8–9 years, followed by the first thinning at 12–13 years and the second thinning at 17–18 years. In addition, they proposed a 25-year rotation (instead of 30 years) in view of the no marked increase in stem diameter/biomass after 18 years. Although Fontalvo-Herazo et al. [33] have used the same observations from Gong and Ong (op. cit.), they found a total gain of 22.95 t ha^−1^ by following the 7-13-20 year old thinning practices in 30 years rotation. According to Fontalvo-Herazo et al. (op. cit.), the 7-13-20 thinning practice would be able to provide best profit possibility for medium sized poles (D_130_: >10 to <13 cm) at those respective managed mangrove forest blocks.

However, the present estimates on tree density, stem diameter, and harvestable biomass are different from the above two investigations. In fact, the increased biomass from 216 t ha^−1^ at MF15 to 317 t ha^−1^ at MF30 (at the rate of 15.5 t ha^−1^ year^−1^) (Figs. 2B-ii-b to 2B-iv-b) coincides well with the earlier estimates [44], and also suggests that the mangrove management based on a 30-year rotation cycle is still appropriate for the MMFR. Moreover, the change in 30 years rotation period was said to be difficult due to commitment of the Forestry Department, mind-set of the pole/charcoal contractors, etc. [35]. As mentioned under the ‘initial stocking’ and the ‘stand density’ sections, the earlier thinning before 15 years is necessary, but the optimal frequency and age of thinning(s) remains uncertain due to no consecutive (field-based) tree structural measurements. The lower rate of biomass increment (0.2 t ha^−1^ year^−1^) between MF15 and MF20, which could be due to limited time interval for the trees (having 73% MST) to grow better after the first artificial thinning, is further adjudicating the necessity of earlier thinning before 15 years. In this context, the increased space of artificial regeneration (see ‘initial stocking’), which ultimately leads to decreased stocking density of the seedlings, might even reduce or compensate the loss of trees at MF15. Therefore a practical validation of the recommendations is warranted.

The mangrove biomass, which varies with age, species and location, is usually higher in the tropics than in temperate areas [64]. When the present biomass estimates are compared with the published literature on natural and replanted *Rhizophora* sites (Table 3), the Matang is contributing a highest biomass for which the ongoing management with a sustainable rejuvenation should also be acknowledged. The trunk biomass, which is principally used for charcoal industry, followed the same trend as that of total above-ground biomass (Figs. 2B-ii-b to 2B-iv-b). In view of the existing differences in stem size distribution, the harvestable biomass was also different between the field- and the simulation-based studies (Fig. 4C).

**Table 3:** A comparative account on the above ground biomass of *Rhizophora* spp. from representative mangrove sites.

	Forest type	Age (years)	Species	Stem diameter (cm)	Mean height (m)	Biomass (t ha^−1^)	Source
Matang, Malaysia	Planted	15	*R. apiculata*	5.0–12.5	12.8	216	Present study
Matang, Malaysia	Planted	20	*R. apiculata*	5.0–15.0	13.3	217	Present study
Matang, Malaysia	Planted	30	*R. apiculata*	15.0–22.5	14.8	372	Present study
Halmahera, Indonesia	Primary	-	*R. apiculata*	-	21.2	357	[74]
Ranong, Thailand	Primary	-	*Rhizophora* spp.	-	-	299	[75]
Yingluo Bay, China	Natural	Oldest	*R. stylosa*	7.4	3.4	271	[76]
Andaman, India	Primary	-	*Rhizophora* spp.	-	22.5	124	[77]
Sri Lanka	Fringe	-	*Rhizophora* spp.	-	7.2	240	[78]
Gazi Bay, Kenya	Planted	5	*R. mucronata*	>5	3.9	20	[79]
Gazi Bay, Kenya	Planted	12	*R. mucronata*	2.5–12.4	8.5	107	[58]
Florida, America	Dwarf	-	*R. mangle*	>2	1.1	12.5	[80]

### Century-long management as a global reference

For efficient conservation and management of the mangrove forests, the cooperation from local people is necessary [2], [65], [66], [67]. Besides the community-based mangrove restoration projects, the locals can be part of carbon financing schemes, eco-tourism development, sustainable sale of commercially valuable timber and non-timber products, etc. [67], [68]. In this context, an appropriate sharing of ‘Revenue, Rights, Responsibility and Relationship’ (4Rs) among the people/stakeholders - whoever interested in that particular mangrove ecosystem, would be able to deliver long-term benefits [8]. The chief objective of the State Forestry Department of Perak is to maximize wood production (for pole and charcoal, both for local use and export) as well as to improve the (local) people's quality of life in the vicinity of Matang [21], [34], [35], [48]. There are as many as 214 mangrove pole and charcoal contractors [35], and every year the MMFR authorities will allocate a minimum 2.2 ha of the productive forest area to each contractor to see their pole and/or charcoal trading are met regularly [35], [69]. This activity is also benefiting several workers under the each contactor for their livelihood.

**Figure 5:**
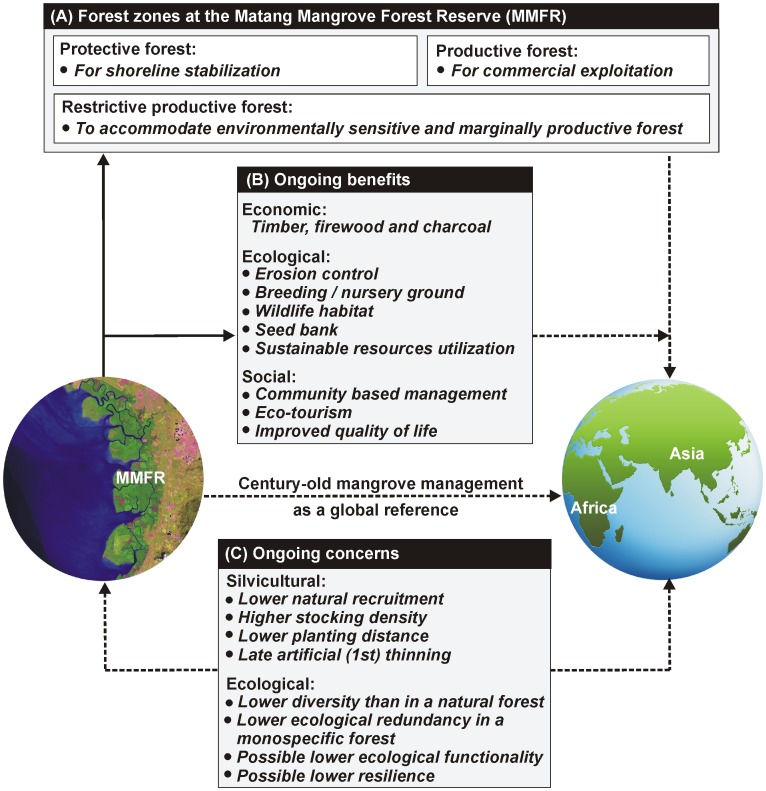
Schematic chart showing the century-old mangrove management at the Matang Mangrove Forest Reserve (MMFR) as a global reference for sustainable silviculture. While the bold-line arrows indicate the features available for Matang (A–B), the dotted-line arrows show the features that could be considered by other mangrove locations for their improved/sustainable mangrove management. Some of the ongoing silvicultural and ecological concerns (C) represented by dotted-arrows, are applicable to the both MMFR and other mangrove locations elsewhere.

In light of the necessity to develop alternative and sustainable energy technologies for personal use and power generation units, the mangrove charcoal was found to be a source of renewable energy [70]. Though it is produced in Thailand, Vietnam and on the east coast of Sumatra of Indonesia, Matang is a unique place where the local people became guardians of the forest for over 100 years and it is also the only example to other mangrove countries for learning/practicing long-term sustainable forest resource utilization [66]. Despite the fact that forest product utilization (in relation to species' availability and local knowledge) varies between the countries [71], [72], the areas with higher human intervention (especially in South and Southeast Asia and African regions) may want to adapt similar guidelines and manage the remaining mangrove forests (Fig. 5). However, the silvicultural management would be more efficient if the given recommendations (with reference to initial stocking, stand density and natural recruitment) are considered by all parties. From the ecological point of view, the VJR and other functional forest categories (i.e. old growth, eco-tourism, education and research) in the vicinity are serving as a mangrove seed bank for Matang as well as other locations in Peninsular Malaysia. The ‘protective’, ‘productive’ and ‘restrictive productive’ forest zones within the reserve are also delivering several eco-socio-economic benefits which are sustainable (Fig. 5).

We also recall that the main emphasis of the MMFR lies on a steady-state resource management of *Rhizophora apiculata* charcoal, which from the ecological perspective is probably less functional and less resilient than a mangrove forest under a resilience-based ecosystem stewardship [59]. For instance, when managing for a single species there is hardly any ecological redundancy in what is naturally already a species-poor ecosystem. Any severe perturbation on a mangrove ecosystem only composed of *R. apiculata* is therefore unlikely to be absorbed by a congeneric species such as *R. mucronata* or even a family representative like *Bruguiera* spp. Although the highly dynamic nature of mangroves generally observed elsewhere [16], [73] may provide an input of propagules from a variety of species, the current management procedure indicates that this is insufficiently the case for MMFR (and also not aimed at). A management aiming at fostering variability within the ecosystem in the light of human-induced and climatic uncertainty and change is therefore lacking. World-wide many exploited and non-exploited mangrove forests suffer severe degradation due to over-exploitation, land conversion, pollution, etc. What MMFR may be exemplary at, is at demonstrating that exploitation does not need to result in degradation, or at least not within a century.

### Conclusions and future perspectives

The present study provided invaluable information for a better conservation and management policy at the MMFR. The results strongly support earlier thinning at the MF15 to reduce the loss of mangrove trees exploitable as fuelwood, both subsistence-based and commercial. At the same time, the reduced initial (stocking) density of the seedlings might be able to compensate additional loss of trees at MF15. Is Matang mangrove forest in Malaysia sustainably rejuvenating after more than a century of conservation and harvesting management? In general, the 30-year rotation cycle with a higher yield of biomass is indeed expected to be sustainable as a silvicultural practice. However, monitoring of wood extraction volumes through socio-ecologic surveys of local cutters should be able to confirm or reject whether or not this expectation is met.

Since the mangrove management plan for 2010–2019 is officially declared, the MMFR authorities may focus on the issues highlighted in this paper (along with their identified research needs), and incorporate necessary changes in the next working plan (i.e. 2020–2029) at least. Meanwhile, the suggestions given for increasing the space of (*R. apiculata*) afforestation as well as time for early thinning(s) could be tested practically by allocating few experimental sites at the MMFR. After witnessing the differences between stem- and tree-based counts, the density calculation/expression should be made appropriately. Despite the fact that MMFR is still an excellent example for sustainable (forest) resources use and wood production in the world, its ecological status and ecosystem services are also in need of urgent and regular monitoring (under appropriate scientific direction), as the functionality in comparison to a natural mangrove may be suboptimal as suggested by the lower diversity in the managed blocks as opposed to VJR. Therefore the term ‘sustainably’ in our title is to be interpreted in a context of silviculture, not necessarily in a context of ecological functionality. A possible solution to maintain an eased recolonization through hydrochory, is to assure a healthy mix between managed blocks (and within management block patches in varied successional stages) and unmanaged blocks. In this way the monospecific managed blocks could bath in a matrix of unmanaged blocks that contain the full local diversity and that can function as a reservoir to renew the adjacent managed blocks after perturbation. This approach may in fact also increase the likelihood of having clear-cut blocks colonised naturally.

The present study/assessment based on silvimetric parameters is of great help not only to the local Forestry Department to manage the resources wisely, but also to the mangrove managers elsewhere who consider Matang as an exemplary for their improved management aiming at silviculture. In addition, the present results are useful to the researchers using mangrove modelling software (e.g. KiWi, Mangal, Forman, NetLogo) for a precise parameterization. The differences encountered between field- and simulation-based studies are due to two different working platforms, limited data for a precise parameterization in mangrove models, different stocking density and reduction percentages. Perhaps a follow-up study to Fontalvo-Herazo et al. [33], integrating present silvimetric observations, would be able to revalidate as well as offer better management options to the MMFR. Meanwhile, our recent findings on the mangrove charcoal production/trade at Matang [69] are also unveiling an eco-socio-economic model for its sustainable long-term management in which wood extraction is matched to the forest's wood productivity, and to the socio-economical sustainability of the mangrove wood trade system in the light of uncertainty and change [Quispe Zuniga, Satyanarayana, Grüters, Berger, Mohd-Lokman, Sulong & Dahdouh-Guebas, unpublished data].
